# Genetic Counseling and NGS Screening for Recessive LGMD2A Families

**DOI:** 10.3390/ht9020013

**Published:** 2020-05-10

**Authors:** Claudia Strafella, Valerio Caputo, Giulia Campoli, Rosaria Maria Galota, Julia Mela, Stefania Zampatti, Giulietta Minozzi, Cristina Sancricca, Serenella Servidei, Emiliano Giardina, Raffaella Cascella

**Affiliations:** 1 Genomic Medicine Laboratory UILDM, Santa Lucia Foundation, 00179 Rome, Italy; v.caputo91@gmail.com (V.C.); GIULIACAMPOLI90@gmail.com (G.C.); rm.galota@gmail.com (R.M.G.); j.mela@hsantalucia.it (J.M.); s.zampatti@hsantalucia.it (S.Z.); emiliano.giardina@uniroma2.it (E.G.); raffaella.cascella@gmail.com (R.C.); 2 Department of Biomedicine and Prevention, Tor Vergata University, 00133 Rome, Italy; 3 Department of Veterinary Medicine, University of Milan, 20133 Milan, Italy; giulietta.minozzi@unimi.it; 4 Fondazione Policlinico Universitario A. Gemelli IRCCS, UOC Neurofisiopatologia, 00168 Rome, Italy; cristinasancricca@me.com (C.S.); serenella.servidei@unicatt.it (S.S.); 5 Unione Italiana Lotta Distrofia Muscolare (UILDM), Sezione Laziale, 00167 Rome, Italy; 6 Department of Biomedical Sciences, Catholic University Our Lady of Good Counsel, 1000 Tirana, Albania

**Keywords:** next-generation sequencing, data analysis, genetic counseling, LGMD2A, *CAPN3*, recurrence/reproductive risk, family history

## Abstract

Genetic counseling applied to limb–girdle muscular dystrophies (LGMDs) can be very challenging due to their clinical and genetic heterogeneity and the availability of different molecular assays. Genetic counseling should therefore be addressed to select the most suitable approach to increase the diagnostic rate and provide an accurate estimation of recurrence risk. This is particularly true for families with a positive history for recessive LGMD, in which the presence of a known pathogenetic mutation segregating within the family may not be enough to exclude the risk of having affected children without exploring the genetic background of phenotypically unaffected partners. In this work, we presented a family with a positive history for LGMD2A (OMIM #253600, also known as calpainopathy) characterized by compound heterozygosity for two *CAPN3* mutations. The genetic specialist suggested the segregation analysis of both mutations within the family as a first-level analysis. Sequentially, next-generation sequencing (NGS) analysis was performed in the partners of healthy carriers to provide an accurate recurrence/reproductive risk estimation considering the genetic background of the couple. Finally, this work highlighted the importance of providing a genetic counseling/testing service even in unaffected individuals with a carrier partner. This approach can support genetic counselors in estimating the reproductive/recurrence risk and eventually, suggesting prenatal testing, early diagnosis or other medical surveillance strategies.

## 1. Introduction

An adequate management of genetic disorders in terms of clinical/molecular diagnosis, prediction of prognosis and possible complications and the estimation of a reliable recurrence risk within a family is essentially based on an accurate and comprehensive genetic counseling. The genetic counseling consists of a number of key elements, such as anamnesis (diagnostic and clinical data of the patient and family documentation), pedigree information, description of the available genetic tests, subscription of the informed consent, explanation of the results and implications of genetic testing, and finally providing support to the decision-making process [1]. During genetic counseling, pedigree analysis is an essential step for assuming a genetic origin for a specific disease, the possible inheritance pattern segregating within the family and to provide information concerning the recurrence and reproductive risks for the family members [1]. However, pedigree analysis may lead to misleading conclusions, especially in cases of incomplete penetrance, genetic heterogeneity, “pseudo-dominant” inheritance pattern and unknown or erroneous familial information. Although pedigree analysis remains crucial in genetic counseling, it is normally complemented with an extensive molecular analysis in order to provide not only the molecular diagnosis (if available) of the disease but also an accurate estimate of the recurrence/reproductive risk and an adequate counseling to the patients. In this context, the genetic counseling applied to disorders characterized by overlapping phenotypes, reduced penetrance and variable expressivity can be very challenging. This is the case of neuromuscular disorders, which include a heterogeneous group of pathologies involving a progressive weakness and wasting of proximal and/or distal muscles [2]. Among them, limb–girdle muscular dystrophies (LGMDs) represent an excellent example of heterogeneous neuromuscular disorders, which primarily affect the proximal limb–girdle muscles [3,4]. LGMDs display more than 30 different subtypes, which have been associated with specific genetic abnormalities [5]. Based on the inheritance model, LGMDs can be classified as autosomal dominant (LGMD1) or recessive (LGMD2). The dominant forms are less common (<10% of total LGMDs) and generally show less severe phenotypes and onset in the adult age. Recessive forms are more frequent (1:15,000), although the prevalence can differ among populations [3]. Among recessive forms, LGMD2A (OMIM #253600, also known as calpainopathy) is the most frequent LGMD worldwide, affecting approximately 30% of all LGMDs cases [6]. The age of onset of LGMD2A is variable, ranging from eight to 15 years of age, although later onset forms have also been described [7]. From a genetic point of view, LGMD2A is caused by mutations located within the *Calpain 3* (*CAPN3*) gene (15q15.2). *CAPN3* is composed of 24 exons encoding the homonymous protein which is primarily expressed in the skeletal muscle [3,6]. *CAPN3* is essential for skeletal muscle regeneration, sarcolemma repair and remodeling, cytoskeleton regulation and calcium homeostasis [8]. To date, approximately 500 different pathogenic *CAPN3* mutations are known and have been found to cause mitochondrial abnormalities, growth failure, increased oxidative stress and sarcomere disorganization [8,9]. 

The molecular diagnosis of LGMDs can be performed by direct sequencing (for known mutations) or next-generation sequencing (NGS, including multigene target-panel, exome and genome analysis methods) [10]. In particular, NGS can be extremely useful to test multiple genes involved in a single or a group of related disorders, to perform a differential diagnosis with similar neuromuscular disorders and to characterize blended or complex phenotypes [11,12,13]. Given the clinical and genetic heterogeneity of LGMDs and the availability of different molecular assays, the genetic counseling should be addressed to select the most suitable approach able to improve the diagnostic rate and provide an accurate estimation of the recurrence risk. In this study, we proposed a workflow for estimating an accurate recurrence risk and providing an adequate pre- and post-test genetic counseling service to families presenting a clinical history for recessive disorders because of the presence of one or more pathogenic mutations segregating in the family members. In fact, the presence of known pathogenic mutations segregating within the family may not be enough to exclude the risk of having affected children without exploring the genetic background of phenotypically unaffected partners. It is important to remark that several pathogenic mutations associated with recessive disorders can be frequently found at the heterozygous state in the general population, meaning that unaffected individuals may be healthy carriers and be at higher risk of having affected offspring if they joined with a carrier partner having a positive family history. The present study took as an example the case of a family with a positive history for LGMD2A characterized by compound heterozygosity for two *CAPN3* mutations. The genetic specialist suggested performing a segregation analysis of both mutations within the family, in order to detect the healthy carriers as a first-level analysis, in accordance to the traditional practice. Successively, the partners of the healthy carriers were subjected to extensive genomic analysis in order to provide an accurate recurrence/reproductive risk estimation based on the genetic background of the couple. This family case was just an example of how the proposed workflow can be useful for providing an adequate genetic counseling to patients concerning their recurrence/reproductive risks and supporting them in their decision-making process. 

## 2. Materials and Methods

The family case was characterized by a family history for recessive LGMD2A, with two subjects being affected and the other family members being phenotypically unaffected. The clinical assessment of the study participants was performed at the Neurophysiopathology unit of the “A. Gemelli” University hospital-IRCSS in Rome, whereas the genetic counseling and testing were taken at the Genomic Medicine Laboratory of Santa Lucia Foundation in Rome. The present study was approved by the ethics committee of Santa Lucia Foundation (CE/PROG.650 approved on 01/03/2018) and was performed according to the Declaration of Helsinki. All participants provided signed informed consent for genetic analysis and in this regard, they provided the consent for the publication of this case report. Genomic DNA was extracted from 400 µL of peripheral blood using a MagPurix Blood DNA Extraction Kit and a MagPurix Automatic Extraction System (Resnova, Rome, Italy) according to the manufacturer’s instructions. The concentration and quality of the extracted DNA was checked by a DeNovix Spectrophotometer (Resnova, Rome, Italy) [14,15].

**Figure 1:**
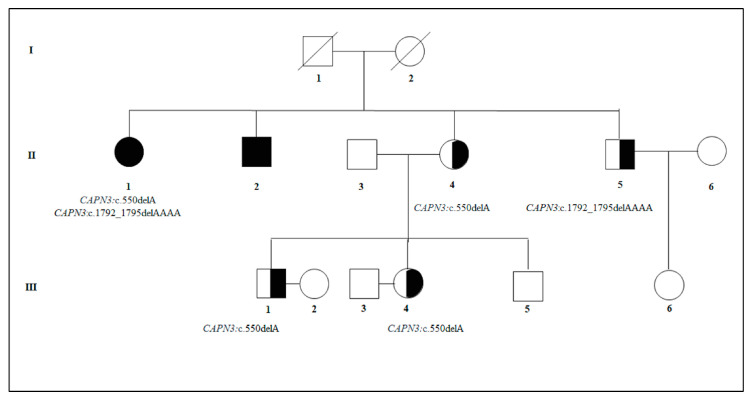
Pedigree showing the segregation of the *Calpain 3* (*CAPN3*)_c.550delA and *CAPN3*_c.1792_1795delAAAA mutations within the family.

The study initially consisted of the direct sequencing of the known *CAPN3* mutations (c.550delA and c.1792_1795delAAAA) segregating within the family (Figure 1). 

The direct sequencing of the samples was performed by a Big Dye Terminator v3.1 Cycle Sequencing Kit (ThermoFisher Scientific, Foster City, CA, USA) and an Abi Prims 3130xl (Applied Biosystems, Foster City, CA, USA). The electropherograms were visualized on Sequencing Analysis Software v.6 (Applied Biosystems, Foster City, CA, USA). 

Successively, the partners of carrier family members were analyzed by NGS in order to screen them for possible pathogenic mutations related to LGMDs, although they did not refer any family history for LGMD2A or other neuromuscular disorders. The NGS sequencing was performed by the Ion PGM System, using an Ion Ampliseq Customized Panel High Specificity, designed by Ion Ampliseq Designer (Thermo Fisher Scientific, Foster City, CA, USA). The panel was expected to screen approximately 99.72% of the target sequences, considering a minimum coverage of 20X. The panel included 18 genes [12], which were selected using scientific literature and GeneReviews [16], as well as the frequency of pathogenic variants in the general population. The libraries’ construction was performed by an Ion AmpliSeq™ Library Kits 2.0. Approximately 10 ng/µL of the starting DNA were utilized for multiplex PCR reactions. Two purification steps (using AMPure XP, Beckman Coulter, Milan, Italy) were performed to remove the unwanted contaminants and successively, a final PCR was set-up according to the manufacturer’s instructions. The quality of the libraries was evaluated by a Qubit^®^ 2.0 Fluorometer. The template amplification and enrichment steps were performed by an Ion PGM Hi-Q OT2 kit-400, Ion OneTouch 2 System and an Ion OneTouch ES (Thermo Fisher Scientific, Foster City, CA, USA). The samples were processed by an Ion PGM Hi-Q Sequencing Kit (400 bp, Thermo Fisher Scientific, Foster City, USA) and run on Ion 316 Chip v2 (850 flows required) and Ion PGM Sequencer (Thermo Fisher Scientific, Foster City, CA, USA). The results were analyzed using an Ion Reporter 5.2 (Thermo Fisher Scientific, Foster City, CA, USA) and an integrated genome viewer (IGV). The interpretation of the genetic variants was conducted by the Human Gene Mutation Database (HGMD), Leiden Open Variation Database (LOVD), ClinVar, 1000Genomes and ExAC. The functional effect of the detected variants was evaluated by bioinformatic predictive tools, such as the Mutation Taster, Varsome, SIFT, PolyPhen 2, SMART, Phyre-2 and the Human Splicing Finder [17,18,19,20,21,22].

## 3. Results and Discussion

This study was mainly focused on the application of genetic counseling to heterogeneous genetic disorders and on the importance of testing asymptomatic individuals in couples with a healthy carrier and a positive history for recessive disorders in order to provide an accurate recurrence/reproductive risk estimation. In fact, it is not possible to exclude that a phenotypically healthy partner is a carrier of a pathogenic allele for a recessive disorder that could increase the chance of having affected offspring (if both partners were carriers). Such a condition is not as rare as expected because some pathogenic alleles for recessive disorders display a high carrier frequency in the general population (for instance, 1:25 for *CFTR* causing Cystic Fibrosis, 1:20 for *ABCA4* associated with retinal dystrophies, 1:103 for *CAPN3* that is responsible of LGMD2A). On this subject, this study presented a workflow for providing an accurate recurrence/reproductive risk estimation based on the genetic and familial background of the couple. As an example, the study reported the case of a family with a positive history for LGMD2A characterized by compound heterozygosity for two *CAPN3* mutations. As a first-level analysis, the family members were tested for the known pathogenic mutations segregating within the family that were *CAPN3*_c.550delA and *CAPN3*_c.1792_1795delAAAA. The segregation analysis in the family case revealed that the siblings (II:4 and II:5) of the affected patients (II:1 and II:2) were heterozygous carriers for *CAPN3*_c.550delA and *CAPN3*_c.1792_1795delAAAA, respectively. The single nucleotide deletion variant c.550delA (p.Thr184Argfs) is the most common pathogenic variant for LGMD among different European Countries, causing 75% of cases [23]. This variant is known to affect protein biosynthesis and function [24]. On this subject, the bioinformatic analysis performed by the Mutation Taster, Phyre 2 and the SMART showed that the variant led to an altered protein with the loss of the essential Calpain-3 and EF-hands domains. The c.1792_1795delAAAA (p.Lys598ProfsTer63) is a homopolymer deletion causing the alteration of protein biosynthesis and structure [25]. In this case, bioinformatic tools (Mutation Taster, Phyre 2 and SMART) predicted the loss of the EF-hand domains.

Given these results, II:4 and II:5 had a 50% of chance of transmitting the pathogenic mutations to the offspring. In fact, the c.550delA was transmitted to the third generation, leading to two unaffected heterozygous carriers (III:1 and III:4) and a wild-type subject (III:5). On the other hand, the c.1792_1795delAAAA was not transmitted to the offspring (III:6), who resulted completely wild type. Given the heterozygous genotype of the III:1 and III:4 subjects and the recessive inheritance model of LGMD2A, we performed the NGS screening on their partners (III:2 and III:3) in order to identify additional LGMD-associated mutations and calculate the reproductive risk for the couples. Both of them were negative to all the 18 tested genes, meaning that they had 1/644 residual risk to be a healthy carrier for LGMD2A-causative mutations, considering that the test was 84% sensitive. The residual risk was obtained taking into account that the probability to be a healthy carrier for a *CAPN3* mutation in the Italian population was 1/103 [26,27] and that 16% of subjects may still carry *CAPN3* genomic alterations although they were negative to the NGS panel. Therefore, given the genetic profile of III:2 and III:3 and the NGS test sensitivity, the residual risk for both couples to have an affected child with LGMD2A was 1/2576. On the basis of these results, the post-genetic counseling for both couples aimed to explain the genetic results and clarify that the risk of having an affected child was actually lower than expected. This work showed the importance of performing genetic counseling not only to affected patients or healthy carriers but also to unaffected individuals with a positive family history for recessive LGMD2A. In fact, asymptomatic patients may still benefit from a NGS-based carrier test that provides them with a comprehensive overview of the individual genetic background and allows a more accurate estimation of the risk of transmitting pathogenic LGMD-associated mutations to the offspring. In addition, the assessment of recurrence and reproductive risks should always consider the prevalence of the disease and carrier status in relation to the specific geographic area of the proband [24]. The presented approach can be widely applied to several cases, not only in relation to LGMDs but also to other recessive disorders. In a previous work, we employed it for estimating the recurrence/reproductive risks in a family case with a positive history of LGMD2A and cardiovascular disease [12]. Concerning the application of the workflow to other genetic disorders, it was regularly utilized in our laboratory for estimating the recurrence/reproductive risk of retinal dystrophies correlated to the presence of pathogenic mutations in different causative genes. This approach was particularly useful in families with a clinical history for retinal dystrophies associated with pathogenic mutations in *ABCA4*. With more than 800 causative mutations, *ABCA4* is responsible for a wide spectrum of autosomal recessive retinal dystrophies phenotypes, including Stargardt disease (STGD1; OMIM #248200), cone–rod dystrophy (CRD), generalized choriocapillaris dystrophy (GCCD) and retinitis pigmentosa (RP) [28]. Moreover, the high carrier frequency (1:20) of *ABCA4* pathogenic alleles in the general population can result in a “pseudo-dominant” inheritance pattern within families [28]. In this context, testing phenotypically unaffected partners of carrier individuals could provide very important information concerning the real risk of having affected offspring. As an example, let us consider the case of a couple with a 30 year-old woman referring a positive family history for retinal dystrophies (the maternal grandmother and a first-degree cousin were affected) and a healthy 35 year-old male without family history for retinal dystrophy or any other ocular genetic disorder. Considering that the family members of the woman have never been tested for the presence of segregating causative mutations, the geneticist suggested performing the NGS screening in the woman, which consisted of the analysis of 24 genes associated with RP and other retinal dystrophies [29]. This analysis allowed identifying the presence of the *ABCA4*:c.5882G>A (p.Gly1961Glu) mutation at the heterozygous state. This mutation (at the homozygous state or in compound heterozygosity with another mutation in *ABCA4*) was frequently associated with retinal dystrophy phenotypes characterized by late onset and a mild phenotype mostly affecting the central part of the retina (macula) [28]. Successively, the specialist suggested performing the same NGS screening in the partner of the carrier woman, in order to exclude any pathogenic mutation in other disease-associated genes and estimate an accurate reproductive risk for the couple of having children affected with *ABCA4*-related disorders. Considering the 80% sensitivity of the NGS test and the carrier frequency (1:20) of *ABCA4* mutations in the general population, the residual risk of being a healthy carrier would be 1:100 for the partner. This means that the couple would have 1:400 residual risk of having an affected child with a retinal dystrophy because of *ABCA4* mutations.

**Figure 2:**
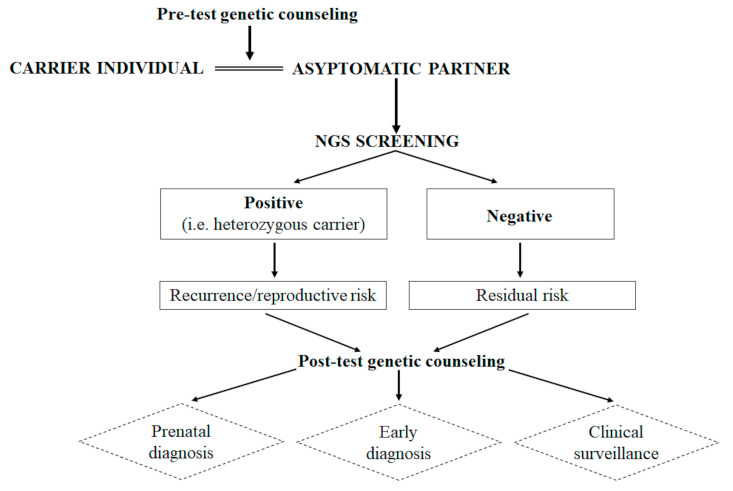
Decision tree describing how the genetic counseling and the testing of asymptomatic individuals can support the medical as well as the patient’s decision-making processes in couples with carrier individuals and a positive family history for recessive disorders such as LGMD2A.

Overall, this study highlighted the importance of providing a genetic counseling/testing service even in unaffected individuals with a carrier partner. Such an approach is helpful for genetic counselors, and can provide a more accurate reproductive/recurrence risk estimation and eventually, suggest the possibility of prenatal testing, early diagnosis or other medical surveillance strategies (Figure 2). Finally, it is important to point out that the clinical significance of genetic counseling does not involve only the single patient, but it also affects born and unborn family members. In this regard, genetic counselors should pay attention to all the possible outcomes and implications of a genetic test at the familial level and thereby provide an accurate genetic counseling.
